# Nitrosative Stress in Retinal Pathologies: Review

**DOI:** 10.3390/antiox8110543

**Published:** 2019-11-11

**Authors:** Antolin Cantó, Teresa Olivar, Francisco Javier Romero, María Miranda

**Affiliations:** 1 Departamento Ciencias Biomédicas, Facultad de Ciencias de la Salud, Universidad Cardenal Herrera-CEU, CEU Universities, 64315 Valencia, Spain; antolin.cantocatala@uchceu.es (A.C.); tolivar@uchceu.es (T.O.); 2 Departamento de Ciencias Biomédicas, Universidad Europea de Valencia, 46010 Valencia, Spain; jromerogomez@gmail.com

**Keywords:** nitric oxide, peroxynitrite, nitric oxide synthase, retina, diabetes, retinitis pigmentosa, glaucoma, age related macular degeneration

## Abstract

Nitric oxide (NO) is a gas molecule with diverse physiological and cellular functions. In the eye, NO is used to maintain normal visual function as it is involved in photoreceptor light transduction. In addition, NO acts as a rapid vascular endothelial relaxant, is involved in the control of retinal blood flow under basal conditions and mediates the vasodilator responses of different substances such as acetylcholine, bradykinin, histamine, substance P or insulin. However, the retina is rich in polyunsaturated lipid membranes and is sensitive to the action of reactive oxygen and nitrogen species. Products generated from NO (i.e., dinitrogen trioxide (N_2_O_3_) and peroxynitrite) have great oxidative damaging effects. Oxygen and nitrogen species can react with biomolecules (lipids, proteins and DNA), potentially leading to cell death, and this is particularly important in the retina. This review focuses on the role of NO in several ocular diseases, including diabetic retinopathy, retinitis pigmentosa, glaucoma or age-related macular degeneration (AMD).

## 1. Introduction

Nitric oxide (NO) is a gas-signaling molecule with a short half-life and diverse physiological and cellular functions [1]. It diffuses across cell membranes and cannot be stored in the human body.

In most cases, NO is synthesized by a reaction in which L-arginine is converted to l-citrulline, with an intermediate reaction product, NG-hydroxy-l-arginine. This reaction is catalyzed by the nitric oxide synthase (NOS) enzyme with the presence of oxygen and nicotinamide adenine dinucleotide phosphate (NADPH) [2]. Vertebrates express three isoforms of the enzyme NOS: i) the constitutive calcium-dependent neuronal NOS (nNOS or NOS-I); ii) the endothelial NOS isoform (eNOS or NOS II) and iii) the inducible or calcium independent NOS isoform (iNOS or NOS III) [3]. Flavin adenine dinucleotide (FAD), flavin mononucleotide (FMN) and (6*R*-)5,6,7,8-tetrahydro-l-biopterin (BH_4_) are cofactors for NOS isozymes [4]. The availability of the substrate and cofactors (NADPH, FMN, FAD, BH_4_) is important for the proper enzyme functionality; a low level of L-arginine or the oxidation of TBH4 protein can cause protein dysfunction.

Typically, eNOS is expressed in vascular endothelial cells; iNOS is mainly associated with inflammation or pathological states, and nNOS is usually expressed in the neurons of the peripheral and central nervous system but also in human skeletal muscles [5]. nNOS and eNOS are constitutive, and nNOS is responsible for the largest proportion of constitutive NOS in humans. iNOS requires induction by immunological factors such as lipopolysaccharide, interferon, and tumor necrosis factor [6]. l-arginine analogs inhibit NOS enzymes. 

An alternative and important source of NO generation is the recycling of nitrates and nitrites. This NO synthesis route is named the nitrate–nitrite–nitric oxide pathway and is particularly relevant in hypoxic states [7].

The only known NO receptor is the enzyme soluble guanylate cyclase. Thus, NO binds guanylate cyclase and increases intracellular cGMP concentrations [7]. Nevertheless, NO may as well be transformed into reactive nitrogen species (RNS) [8]. The term nitrosative stress describes the ability of NO to react with molecules such as proteins and DNA, and these reaction products may be harmful for the cells [9].

**Figure 1:**
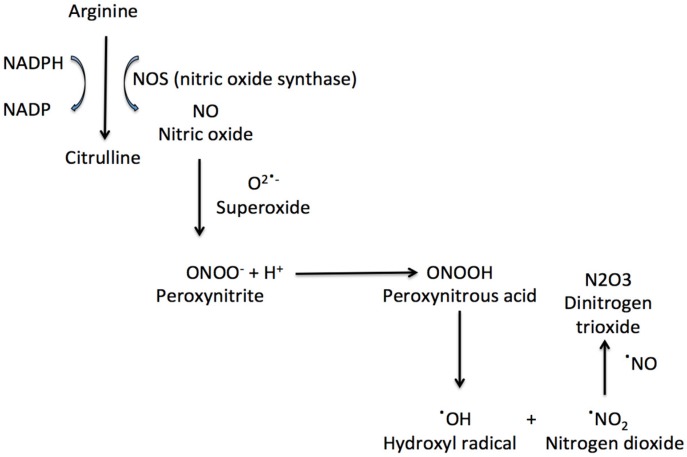
The synthesis of nitric oxide and some important nitrosative species.

NO can react with superoxide anions to form peroxynitrite (ONOO). ONOO formation induces cytochrome c release, which can induce cell death and which blocks the respiratory chain [8]. Peroxynitrous acid (ONOOH), peroxynitrite conjugate acid, reacts with nitrate without forming strong oxidant intermediates. ONOOH can also form OH and nitrogen dioxide, which can initiate fatty acid oxidation and amino acids nitration (Figure 1). The peroxynitrite reactivity is also affected by carbon dioxide, with the formation of a reactive nitrosoperoxocarbonate [10].

NO can also react with proteins. S-nitrosation is a reaction of the NO moiety with the low-molecular thiols or cysteine amino acid residue of proteins to form S-nitrosothiols. An important intermediate reaction product in nitrosation is S-nitrosoglutathione [9]. Nitration is a chemical reaction resulting in the formation of products such as 3-nitrotyrosine [9]. Both the products of S-nitrosation and nitration may damage the cell. The dysregulation of nitrosation and nitration has been linked to human neurodegenerative disorders and is mostly related to the excessive production of NO that takes place through the excessive nNOS or iNOS activity via neuroinflammatory stimuli or several toxin actions. [11,12]. Oxidative stress has been shown to convert eNOS from an NO-producing enzyme to an enzyme that generates O_2_^•−^. This process has been referred to as NOS uncoupling [4] and may be important in vascular diseases, such as diabetic retinopathy.

## 2. Nitric Oxide in the Retina

Different retinal regions present an NO production. nNOS expression has been found in the inner retina [13]. The nNOS isoform has been located anatomically by immunohistochemistry in the mouse retina in the outer plexiform layer (OPL), in bipolar, amacrine and ganglionar somas and in cellular processes in the internal plexiform layer (IPL) [14,15,16,17]. Studies of the expression of different NOS isoforms in mouse retinas suggest that the immunoreactivity for nNOS reflects most of the production of NO [15].

iNOS is not constitutively expressed; its expression is inducible by pro-inflammatory stimuli. It is known that iNOS is expressed in the inner nuclear layer (INL) in an in vivo murine model of proliferative ischemic retinopathy where there is marked damage to the internal retina [18]. Other authors have shown that, after infection of the retina by a murine cytomegalovirus, most cells that express iNOS in the retina are macrophages and microglia [19]. The presence of iNOS in the outer segments of the photoreceptors has been demonstrated in the retinal degeneration rd2 mouse [20]. iNOS may be also important in the normal phagocytosis of the retinal outer segment, in ischemic processes and in diabetic retinopathy pathogenesis [6]. 

eNOS expression in endothelial cells has even been detected in the human fetal eye [21]. eNOS immunoreactivity has also been demonstrated in photoreceptors, horizontal cells, bipolar cells, amacrine cells, Müller cells, and in the ganglion cell layer of the avian retina [22]. After retinal injuries in rats, such as ischemia/reperfusion, it has been shown that eNOS immunoreactivity increased in retinal vessels and in the ganglion cell layer [23]. 

Regarding the retinal NO function, NO is used to maintain a normal visual function. It is involved in photoreceptor light transduction, as 3’,5’cyclic guanosine monophosphate (cGMP) production, an essential intermediate in the visual transduction cascade, is catalyzed from GTP by guanylate cyclase, a target enzyme for the action of NO [24]. In addition, NO acts as a rapid vascular endothelial relaxant and is involved in retinal blood flow control under normal circumstances, and it mediates the vasodilator responses of different substances such as acetylcholine, bradykinin, histamine, substance P or insulin [25].

However, the retina is rich in polyunsaturated lipid membranes and is especially sensitive to the action of reactive oxygen and nitrogen species [3]. Despite NO itself being a radical, its reactivity is low compared to the possible damage generated by the oxidative products that it generates, such as dinitrogen trioxide (N_2_O_3_) and peroxynitrite (ONOO−). Both can react with biomolecules, which can lead to cell death [26]. In this sense, NO has been related to several ocular diseases, including diabetic retinopathy, retinitis pigmentosa, glaucoma or age-related macular degeneration (AMD) [26].

## 3. Diabetic Retinopathy

Diabetic retinopathy (DR) is one of the leading causes of blindness in adults in developed countries. DR is categorized into non-proliferative DR (NPDR) and proliferative DR (PDR) phases according to the presence of visible ophthalmologic changes and evidence of retinal neovascularization [27].

NPDR is a consequence of hyperglycemia, which accompanies these patients and weakens the capillary walls, leading to the formation of microaneurysms. This is followed by the rupture of vessels, leading to lipid by-products deposition. In addition, the nerve fibre layer may be obstructed, resulting in the accumulation of white spots or cotton wool spots [27]. NPDR is followed by PDR, which is characterized by neovascularization. These newly-formed vessels are leaky and fragile. They may also lead to hemorrhages and tractional retinal detachment. Macular oedema, the last stage of DR, is the principal cause behind the loss of visual acuity [27].

As we have just described, DR has been classically considered a microvascular disease. However, increasing evidence suggests that retinal neuron death occurs before vascular changes. In this sense, DR can now be described as a microvascular disease but also as a neurodegenerative disease [28].

Although various biochemical pathways may be the basis of the DR aetiology, the main insult to the retina is due to oxidative stress and inflammation [27]. Hyperglycaemia and other stress stimuli (including oxidative stress) trigger the generation of superoxide, which reacts with NO, producing peroxynitrite. Peroxynitrite can lead to cell damage. A question that has not yet been completely answered is which of the three NOS isoforms is responsible for the damages observed in retinal cells during DR.

In 2010, Li et al. [29] induced diabetes by streptozotocin injection in eNOS knockout (−/−) mice. Their results showed that eNOS −/− mice exhibited a more severe retinal vascular permeability than control mice did. eNOS −/− mice showed an earlier and increased number of acellular capillaries, increased capillary basement membrane thickness, gliosis and total NO-relative products (assessed by measuring nitrate/nitrite using a fluorometric-based assay). The authors suggested that this NO concentration increase was due to the elevated iNOS expression in the diabetic eNOS −/− retina [29].

NOS requires a physiologically relevant amount of L-arginine to produce NO. When L-arginine is limited, NOS becomes uncoupled, causing it to produce superoxide that will react with NO to form ONOO^−^ and reducing NO. This NO decrease may be responsible for the diabetes-induced decreases in the retinal blood flow that have been observed in diabetic humans and DR animal models [30]. It is well known that a physiologically relevant production of NO by eNOS is necessary for the maintenance of healthy vessels and a proper blood flow.

Other studies had focused on the relevance of iNOS in DR. Zheng et al. [31] induced diabetes in control and iNOS −/− mice and found that the retinas were thinner, that the number of acellular capillaries and pericyte ghosts increased in the retinas of diabetic mice and that these changes were not observed in iNOS −/− mice. However, the deletion of iNOS had no effect on the diabetes-induced abnormalities usually observed by means of an electroretinogram.

In humans, Sharma et al. [32] studied 60 diabetic patients and classified the severity of their DR according to an early treatment diabetic retinopathy study (ETDRS) classification. Their study demonstrated that increased NO plasma concentrations are associated with an increased diabetic retinopathy severity. The aqueous humour NO concentration has also been determined in type 2 diabetic patients that were subjected to cataract surgery, and was found to increase when compared with control patients [33].

In diabetic human post-mortem retinas, immunoreactivity for iNOS has been detected in ganglion cells, glial cells and cells of the INL, and nitrotyrosine immunoreactivity has been detected in vascular endothelial cells [34]. In retinas from subjects without diabetes, there was no iNOS or nitrotyrosine immunoreactivity [34].

### Can NO Inhibition Have a Role in Diabetic Retinopathy Therapy?

A great number of studies have shown that the antioxidant administration in animal models of diabetes can reduce retinal cell death by apoptosis, and can thus contribute to the improvement in DR progression [35,36,37]. However, the results of clinical trials that have used antioxidants in DR therapy are inconclusive [38]. Consequently, new studies and approaches using antioxidants as adjuvant therapies in DR treatment should be performed. In this sense, the possible use of antioxidants and/or NOS inhibitors and NO scavengers has been suggested [39]. It has been reported that aminoguanidine, a pharmacological inhibitor of iNOS and an inhibitor of advanced glycated end products, prevents the histological changes induced by diabetes in rats and that it is safe when administered intravitreally [40]. Other NOS inhibitors, such as N(G)-nitro-l-arginine methyl ester (l-NAME), are able to reduce the increase in oxidized proteins observed in diabetic rat retinas [41]. Moreover, it is known that in diabetic retinas, iNOS and the bradykinin type 1 receptor contribute to inflammation, oxidative stress, and vascular dysfunction and that the administration of a selective iNOS inhibitor for diabetic rats decreases these alterations [42]. Conversely, a large septic shock trial was terminated prematurely because of a trend toward harm among patients receiving NO inhibitors [43]. 

Further studies are needed in order to know which of these molecules may be useful and to determine their optimal doses, toxicity, administration form, etc.

## 4. Retinitis Pigmentosa

Retinitis pigmentosa (RP) is a group of retinal hereditary pathologies that occurs in 1 out of 4000 people worldwide. RP mutations cause rod cell death via several different mechanisms. These changes result in a progressive loss of the visual field and an abnormal electroretinogram [44,45]. This loss of the peripheral vision is accompanied by an alteration of the arterial supply of the retina, the precipitation of bone spicules (residual granules of the pigment metabolism) between the pigment cells and around the vessels, and a pale optical disc [44,46]. In last place, the pathology progression affects the cones [44,45]. An electroretinogram in the initial RP stages manifests a scotopic vision loss related to the death of rods without an apparent change in the fundus. As the disease progresses and the cones are affected, the photopic vision is also affected [44,47].

The main RP symptoms are initially a difficulty of adaptation in the scotopic vision and a loss of the middle and peripheral visual fields, followed by a loss of the central visual field [47].

The genetic basis of the disease is usually monogenic; however, these are very heterogeneous. More than 45 genes related to the disease are known, including the rhodopsin gene that causes 25% of the dominant RP cases, the USH2A gene that causes 20% of the recessive cases and the RPGR gene which causes 70% of cases of retinitis pigmentosa linked to chromosome X [47].

RP has been related to oxidative stress [48,49,50,51,52], not only while rods die but also when cones are secondarily dying. Rods degeneration decreases oxygen consumption in the retina, inducing a large excess of oxygen in the outer retina [53]. The oxygen excess’s main consequences are an excess of superoxide radicals and progressive oxidative damage to the cones [54,55]. Due to the high nitric oxide levels in the retina, free radicals generate peroxynitrite, which is extremely reactive and difficult to detoxify.

Our group has demonstrated a decrease in iNOS and no significant change in nNOS in the retina of rd10 mice, an RP animal model [56]. These results agree with Yang et al., who in 2007 studied the expression of iNOS in the rd2 mice (another RP mice model). These authors observed that in the retina of control mice there was a constitutive expression of iNOS. Moreover, the expression of iNOS was regulated in a time-dependent manner due to degeneration. It increased from postnatal day 12 to 14 and decreased after these post-natal days [20]. 

**Figure 2:**
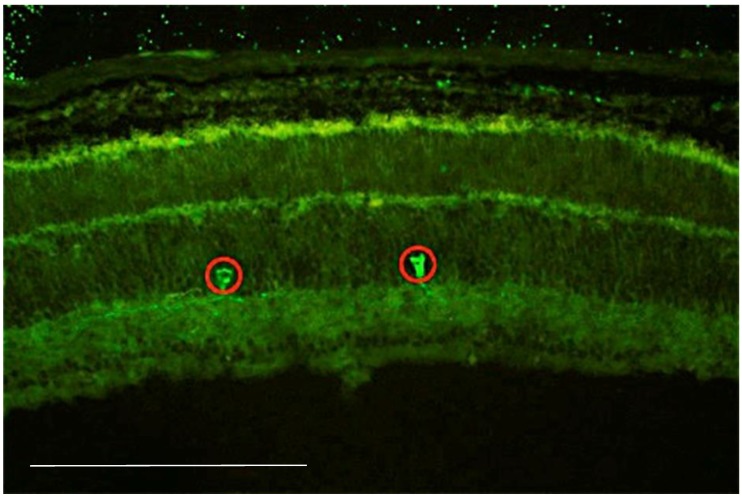
Image of a retina from a postnatal 11-day rd1 mouse, showing immunostaining for neuronal nitric oxide synthase (nNOS). Immunofluorescent staining was performed on retinal cryosections that were incubated overnight with the primary antibody anti-neuronal nitric oxide synthetase (anti-nNOS) (1:200, Santacruz Biotechnology, Dallas, USA). The sections were later incubated with the fluorescence-conjugated secondary antibody Alexa Fluor 488 (Invitrogen, Life Technologies, Madrid, Spain). (Scale: 200 μm).

Interestingly, the mutation and the disease course in rd1, rd10 and rd2 mice (three different RP animal models) is very different. The mutation in the rd1 mice is recessive and is located on chromosome 5 of the rod cGMP-specific 3’,5’-cyclic phosphodiesterase subunit beta (PDE6β) gene. This mutation results in a severe and early degeneration of the retina due to the insertion of a murine virus that introduced a nonsense mutation in the 7th sense of the gene. Two characteristics resulting from the mutation have been described: a deficiency in the catalytic activity of PDE6β and a subsequent accumulation of cyclic guanosine monophosphate (cGMP) [57]. The mutation in the rd10 mice is also located on chromosome 5, and it is induced by a missense mutation in exon 13 of the PDE6β gene [58]. The retinal degeneration in the rd10 mouse model is not caused by the absence of the PDE6β protein but rather by a deficient expression and/or low activity of this enzyme, which could lead to an accumulation of cGMP, more slowly but similar to that found in the rd1 retina [58]. Our group has demonstrated an increase in retinal glutamate concentrations both in rd1 and rd10 mice [51]. This increase may explain the role of NO in this RP animal model because glutamate stimulates N-methyl-d-aspartate (NMDA) receptors, increasing the intracellular Ca^2+^ concentration and the production of NO [6]. Our group has also demonstrated the expression of nNOS, mainly in the soma and axons of amacrine cells (Figure 2) in the retina of rd1 mice but have failed to demonstrate any increase or alteration of this enzyme expression in the RP mice.

The rd2 retinal degeneration model has a spontaneous dominant mutation in the peripherin 2 protein (Prph2). The photoreceptor cells in these mutants do not have outer segments that should start to develop at 7 days of age. The mutation is an insertion of foreign DNA into an exon of Prph2, which causes the transcription of an abnormally large mRNA [59]. Peripherin 2 encodes a membrane protein that is located in the outer segment of photoreceptor cells and is involved in the disc photoreceptor morphogenesis. It may be interesting to determine if there is an increase in the retinal glutamate concentration in this animal model.

### Retinitis Pigmentosa Treatment with Nitric Oxide Synthase Inhibitors

Metipranolol, an antiglaucoma and antihypertensive drug, slowed the rod and cone cell death in the rd10 model of RP [60]. This effect may be mediated by its ability to inhibit nitrosative stress [59]. The treatment of rd1 mice with a mixture of nitric oxide synthase inhibitors (including NG-nitro-l-arginine, Nω-nitro-l-arginine methyl ester, N-monomethyl-l-arginine and aminoguanidine bicarbonate) reduced S-nitrosocysteine and nitrotyrosine staining and improved cone survival [61]. In this study, a specific inhibitor of neuronal NOS was also able to decrease cone cell death, while a specific inhibitor of inducible NOS was not able to increase cone survival [61].

## 5. Glaucoma

Glaucoma is considered the first cause of irreversible blindness in the world. It is believed that the number of people with glaucoma worldwide will increase to more than 100 million in 2040 [62]. Intraocular pressure (IOP) is considered the main risk factor of glaucoma. IOP is determined by the balance in aqueous humour production by the ciliary epithelium and the elimination through the conventional trabecular meshwork (TM) system and the uveoscleral tract. 

Glaucoma can be classified into several types: i) primary open-angle glaucoma (POAG), which is characterized by high IOP, damage of the optic nerve and visual field loss; ii) primary angle-closure glaucoma (PACG), characterized by the occlusion of the trabecular meshwork by the peripheral iris obstructing aqueous outflow and an elevated IOP that can cause damage to the optic nerve head and retinal ganglion cell death; and iii) normal-tension glaucoma (NTG), which is considered a variant of POAG [63].

It makes sense to assume that the alterations in NO and its metabolism may be related to the pathogenesis of glaucoma. TM consists of smooth muscle-like cells, and their ability to relax in response to NO is known, as a consequence of which the outflow resistance and IOP may decrease [64]. In addition, NO may also prevent glaucoma symptoms because of its capacity for vasodilation, which can improve retina and optic nerve head perfusion [65].

The levels of NO in the vitreous humour have been determined in humans with different glaucoma types and in animal models; however, the results are contradictory. Doganay et al. found lower humour NO concentrations in patients with POAG than in patients with cataracts and no glaucoma, concluding that cells that produce NO may be lost with the disease progression [66]. However, others found that the aqueous humour NO level varied with different glaucoma types and that these levels were higher in glaucoma patients than in cataract patients [67]. 

It has also been suggested that glutamate toxicity is related to glaucoma pathogenesis and that it is elevated in the vitreous humour of animal models of glaucoma and of glaucoma patients [68].

NOS and other enzymes related to the NO metabolism may also be affected depending on whether the patient has glaucoma and on the severity of the disease. NADPH diaphorase (known to colocalize with eNOS) immunostaining has been found to decrease in several eye tissues, such as TM, Schlemm´s canal or the ciliary muscle in 12 POAG patients when compared to 10 patients without glaucoma [69]. There is also evidence of the association between variants in the endothelial nitric oxide synthase gene and POAG in women, though this could not be assessed in men [70]. Likewise, Pang et al. demonstrated that glaucoma was not associated with a significant change in the iNOS immunoreactivity in the retina and optic nerve of humans and in a rat optic nerve damage model [71]. 

It may be concluded that NO can have protective or toxic effects in glaucoma patients depending on the concentration. In this sense, it has been suggested by Toda and Nakanishi that NO normally formed in the eye is advantageous in at least preventing glaucoma development, but an excessive activation of iNOS, with the consequent increase in NO levels, is detrimental for glaucoma patients [72].

### Nitric Oxide in Glaucoma Treatment

Despite previous observations, it has been suggested that targeting glaucoma with NO compounds can be helpful for improving glaucoma symptoms, such as the malfunction of the TM outflow or the increase in IOP [73]. Indeed, NO donors are used in other diseases, including myocardial infarction and bacterial infections. Moreover, studies with glaucoma patients who had taken nitroglycerin (for non-ophthalmic reasons) demonstrated that these patients were better protected against this optic nerve pathology [73]. 

Regarding ocular administration, the two most studied drugs related to NO that have demonstrated good results in glaucoma patients are latanoprostenebunod and nipradilol, the first one being a combination of latanoprost with a NO-donating molecule and the second one being an NO donor [72].

## 6. Age-Related Macular Degeneration

Age-related macular degeneration (AMD) is the leading cause of blindness in the elderly in developed countries. It is thought that people with AMD in 2020 will number 196 million, increasing to 288 million by 2040. Cases of AMD are less prevalent in Asia than in North America and Europe, and there is no difference in the gender prevalence [74].

AMD induces the irreversible destruction of the macula, which causes the loss of sharp and detailed vision [75]. It is classified into two forms: i) neovascular or wet AMD, and ii) non-neovascular or dry AMD. In the neovascular AMD, there is a rapid and severe loss of vision, mainly due to the uncontrolled growth of new blood vessels under the macula, which can leak fluid and induce haemorrhages and fibrosis [76]. Non-neovascular AMD is characterized by the presence of lipid yellowish deposits called drusen, retinal pigment abnormalities and subretinal deposits (pseudodrusen). These abnormalities can cause retinal pigment cell and photoreceptor death, as well as gradual vision loss [76]. The AMD stages can also be classified as early, intermediate and late stages [75].

Over the past 15 years, anti-vascular endothelial growth factor (VEGF) treatments have improved the vision preservation and quality of life of a great number of patients with wet or neovascular AMD [75]. However, no effective treatment is available for dry or non-neovascular AMD patients, and new innovations are also needed for wet AMD treatment [75,76]. In order to find new AMD treatments, it is important to know the exact etiopathogenesis of this disease.

Although the exact mechanism of AMD is still unknown, it has been established that advanced age is the main risk factor but that other factors could also be involved. These factors include cigarette smoking, elevated cholesterol levels, systemic arterial hypertension and stiffness, ultraviolet exposure, cardiovascular disease, race, gender and family history [77].

A vascular theory, that includes abnormalities of the choroidal circulation has been suggested to contribute to AMD development [78]. Even though, the precise role of NO in the onset of AMD has not been clearly determined, the involvement of oxidative stress and NO as a part of the vascular theory has been reported [77]. Both factors (oxidative stress and NO alterations) can contribute to the vascularization decrease of choriocapillaris and apoptosis, to the increasing formation of drusen and to the increased VEGF release from the retinal pigment epithelium cells [79].

Lower NO and higher malondialdehyde (MDA) concentrations have been found in the plasma of AMD patients compared to control patients [79]. The authors suggest that an increase in oxidative stress can contribute to a decrease in NO because of the formation of peroxynitrite. This could lead to the inhibition of the NO effects on the regulation of the ocular flow and the induction of a further reduction of NO production by eNOS [80].

NOS expression has also been found in AMD eyes. In this sense, it has been shown that nNOS expression decreased in retinal ganglion cells, retinal vessels and the retinal pigment epithelium of AMD eyes, when compared to control patients; furthermore, eNOS expression was also significantly decreased in choroidal arteries and cells [81]. 

Other researchers have suggested that retinal ischemia that occur as a result of AMD can induce an increase in NO production [26]. Ischemia produces increases in glutamate and aspartate, leading to increases in intracellular calcium and free radical oxygen activity, which induces cell death and an increase in NO production by nNOS. iNOS can also form more NO, because this enzyme is also stimulated by the inflammation found in tissues with increased oxidative stress [26].

### Treatment Perspectives of Age-Related Macular Degeneration with Molecules Related to Nitric Oxide Metabolism

As in other retinal diseases, it seems that NO consequences are related to its concentration and to the enzyme that is responsible for its production. A normal NO retinal concentration is essential for vision processing, but an excess is detrimental. In a similar manner, eNOS-derived NO plays an important role in the ocular blood flow regulation, but iNOS-derived NO could be responsible for the increase in reactive nitrogen species and may cause death [79]. In conclusion, to stimulate one type or other of NOS is important when designing new possible AMD treatments.

## 7. Conclusions

Though new therapies targeting nitrosative stress may be effective for numerous retinal pathologies, further studies are needed. NO is essential for normal vision, but an excess of NO may lead to complications in retinal pathologies.
